# Vascular stability of brain arteriovenous malformations after partial embolization

**DOI:** 10.1111/cns.14136

**Published:** 2023-02-27

**Authors:** Yingkun He, Yanyan He, Weixing Bai, Dehua Guo, Taoyuan Lu, Lin Duan, Zhen Li, Lingfei Kong, Juha A. Hernesniemi, Tianxiao Li

**Affiliations:** ^1^ Cerebrovascular and Neurosurgery Department of Interventional Center Zhengzhou University People's Hospital, Henan Provincial People's Hospital Zhengzhou China; ^2^ Henan Provincial Neurointerventional Engineering Research Center, Henan International Joint Laboratory of Cerebrovascular Disease and Henan Engineering Research Center of Cerebrovascular Intervention Zhengzhou China; ^3^ Department of Pathology Zhengzhou University People's Hospital Zhengzhou China

**Keywords:** angiogenesis, brain arteriovenous malformation, intracerebral hemorrhage, partial embolization

## Abstract

**Introduction:**

Brain arteriovenous malformation (bAVM) might have a higher risk of rupture after partial embolization, and previous studies have shown that some metrics of vascular stability are related to bAVM rupture risk.

**Objective:**

To analyze vascular stability of bAVM in patients after partial embolization.

**Methods:**

Twenty‐four patients who underwent partial embolization were classified into the short‐term, medium‐term, and long‐term groups, according to the time interval between partial embolization and surgery. The control group consisted of 9 bAVM patients who underwent surgery alone. Hemodynamic changes after partial embolization were measured by angiogram. The inflammatory infiltrates and cell–cell junctions were evaluated by MMP‐9 and VE‐cadherin. At the protein level, the proliferative and apoptotic events of bAVMs were analyzed by immunohistochemical staining of VEGFA, eNOS, and caspase‐3. Finally, neovascularity and apoptotic cells were assessed by CD31 staining and TUNEL staining.

**Results:**

Immediately after partial embolization, the blood flow velocity of most bAVMs increased. The quantity of MMP‐9 in the medium‐term group was the highest, and VE‐cadherin in the medium‐term group was the lowest. The expression levels of VEGFA, eNOS, and neovascularity were highest in the medium‐term group. Similarly, the expression level of caspase‐3 and the number of apoptotic cells were highest in the medium‐term group.

**Conclusion:**

The biomarkers for bAVM vascular stability were most abnormal between 1 and 28 days after partial embolization.

## Graphical abstract

Partial embolization plays a vital role in the management of bAVM. However, the optimal interval between nidal resection and preoperative adjuvant partial embolization in bAVM patients is still controversial. In addition, the prognostic impact of partial embolization on patients with bAVM has not been established. Our study is the first to systematically describe the changes in vascular stability biomarkers of bAVM after partial embolization. We provide the first evidence that these vascular stability‐related indexes are dynamic, varying with the time between embolization and resection. And, we first find that the biomarkers for bAVM vascular stability were most abnormal between 1 and 28 days after partial embolization. This may be a plausible explanation for the highest risk of rupture in the relatively early period after partial embolization in patients with bAVMs. Based on our results, we first provide a molecular biology level of evidence that support the viewpoint that resection surgery after partial embolization of bAVM patients should be performed early to avoid the high rupture risk period after partial embolization.

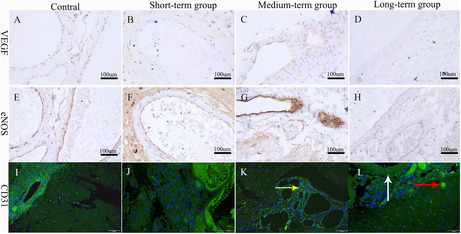

## 1 INTRODUCTION

Brain arteriovenous malformation (bAVM) is characterized by a “nidus” of vascularity lacking a capillary bed between the cerebral arteries and veins. bAVMs can cause intracerebral hemorrhage (ICH), epilepsy, and neurological deficits. Combined treatments are often necessary including microsurgery, radiosurgery, and endovascular embolization.^1^

Partial embolization plays a vital role in the management of bAVM, especially for patients with high‐grade bAVMs. Such therapies may be employed to eliminate high‐risk features, reduce the steal phenomenon, reduce blood flow in preparation for surgical resection, as well as modify lesion size in preparation or radiosurgery. Prior research has suggested that partial embolization can cause the proliferation of nidal vascular tissue, which may increase the risk of bAVM rupture.^2^, ^3^ A 2‐year follow‐up survey showed that the risk of bAVM rupture reached the highest in the first year after partial embolization.^4^ Therefore, all current guidelines, consensus do not recommend partial embolization alone as the first treatment for patients with bAVMs. However, Aki Laakso et al.^5^ at their 30‐year follow‐up found that patients with partially occluded *bAVMs* had worse relative survival within 1–2 years compared with conservatively managed patients, however, this trend reversed after year 5. This suggests that the effect of partial embolization on the patient may be dynamic. To date, few studies have concentrated on the effects of partial embolization on the bAVM nidus over time, especially at a molecular level. Investigating the specific changes in bAVMs after partial embolization could help to search for factors that cause bAVM rupture after partial embolization.

At present, the known triggers for bAVM rupture include hemodynamic stress, inflammatory infiltration, high vascular leakiness, and exuberant neovascularization, that is, the bAVM vessels are unstable.^6^, ^7^, ^8^ In the present study, we investigated the association between partial embolization and vascular stability of bAVMs.

## 2 METHODS

### 2.1 Sample collection

Tissue specimens of bAVM, which had been resected by the senior author (J. A. H.), were retrospectively obtained from Department of Pathology, Henan Provincial People's Hospital. This study was approved by the Ethics Committee of Henan Provincial People's Hospital (Zhengzhou, China). All patients had given written informed consent. To ensure the antigenic validity of the samples (preserved time <3 years), we only enrolled the patients who underwent bAVM resection between June 2018 and January 2021. In total, 45 bAVM tissue specimens were obtained from the department of pathology. Five samples were either too small or of too poor a quality to be included in our analysis. Propensity score‐matching analysis was used to reduce the effect of selection bias between the surgery group and the preoperative partial embolization group. For propensity score matching, we used Spetzler–Martin grade as the covariate,^9^ and a nearest‐neighbor 1:1 matching scheme with a caliper size of 0.02 was used. Finally, 33 patients were enrolled, and 24 patients underwent preoperative partial embolization and nine patients underwent surgery alone. The embolization strategy of 24 patients include palliative embolization, reduced blood flow for resection, or targeted embolization to exclude the high‐risk features such as intra‐nidal aneurysm or fistulas (one patient received embolization of an intranidal fistula). These 24 patients were grouped according to the time between partial embolization and surgery, including the short‐term group (interval <1 day, *n* = 9), medium‐term group (1 day <interval <28 days, *n* = 8), and long‐term group (interval >28 days, *n* = 7). Patients' characteristics are presented in Table 1.

**TABLE 1:** Clinical characteristics of subjects providing tissue samples.

Parameter	Control	Short‐term	Medium‐term	Long‐term	*p*‐Value*
No. of specimens	9	9	8	7	
Mean age in years	29.3 ± 16.7	26.7 ± 18.6	28.3 ± 12.2	25.9 ± 8.8	0.902
Sex					
Male	7(77.8)	5(55.6)	3(37.5)	3(42.9)	0.374
Female	2(22.2)	4(44.4)	5(62.5)	4(57.1)	
Lesion location					
Supratentorial	8(11.1)	8(11.1)	8(100)	6(85.7)	1.0
Infratentorial	1(88.9)	1(88.9)	0(0)	1(14.3)	
Spetzler–Martin grade					
I	0(0)	0(0)	0(0)	0(0)	0.244
II	5(55.6)	3(33.3)	1(12.5)	1(14.3)	
III	4(44.4)	3(33.3)	3(37.5)	4(57.2)	
IV	0(0)	3(33.3)	4(50.0)	2(28.6)	
V	0(0)	0(0)	0(0)	0(0)	
Ruptured history					
Ruptured	7(77.8)	7(77.8)	6(75)	7(100)	0.602
Unruptured	2(22.2)	2(22.2)	2(25)	0(0)	
Mean sugery interval (day)	—	0.255 ± 0.14	6(4,13)	2190(120,3285)	—

*Note*: Angiography after embolization showed residual deformity in all cases. The embolization agent used was Onyx. In the short‐term group, the shortest time interval was immediately after embolization and the longest time interval was 9 h; in the medium‐term group, the time interval was 3–21 days, and in the long‐term group, the interval time was >85 days. — = not applicable. Values are expressed as the mean ± standard deviation, median (P25,P75), or as number (%).

* Comparison between groups.

### 2.2 H&E staining

All 33 bAVM tissues were sectioned into 5 mm slices, followed by deparaffinization and rehydration. H&E staining was performed according to the instructions of a kit (Beyotime, Shanghai, China). The pathological features of tissues were observed under a light microscope. Then, the observed pathological features were compared with prior publications.

### 2.3 BAVM blood flow analysis

All patients had at least one pre‐operative digital subtraction angiography (DSA) at our hospital performed at 6 frames/s. Nidal blood flow velocity was defined as the number of interval frames appearing between the first feeding artery and the first draining vein, and the number of interval frames were manually calculated according to DSA images, and all images from the DSA record were included in the calculation. For patients who underwent preoperative partial embolization, the number of interval frames after embolization of each feeding artery was manually calculated. In addition, the changes in the number of interval frames for patients who underwent repeat DSA before surgery were also calculated. The calculations were performed by three experienced neurointerventional surgeons.

### 2.4 Immunofluorescence and TUNEL staining

The microvessel density in bAVM tissues was evaluated based on the CD31 staining. The cell–cell junctions and inflammatory infiltration were detected by double‐immunostaining of VE‐cadherin and MMP‐9 antibodies. The apoptotic cells were counted by TUNEL staining. The experimental details can be found in the Supplemental Digital Content.

### 2.5 Immunohistochemistry

VEGFA, eNOS, and caspase‐3 were quantified by immunohistochemistry, and the experimental details can be found in the Supplemental Digital Content.

### 2.6 Statistical Analysis

The Shapiro–Wilk test was used to assess the normality of all continuous data. Continuous data are presented as mean ± SD or median (P_25_,P_75_) as appropriate. Levene‘s test was used to assess the homogeneity of variance for all normally distributed data. According to the results of the normality test and variance homogeneity test, all continuous data were analyzed with one‐way analysis of variance (AVOVA) or the Kruskal–Wallis test to determine differences between groups. The changes in interval frames before and after partial embolization were calculated using the paired‐samples nonparametric test. For categorical variables, Fisher's exact test was employed to analyze differences between the two groups. *p* < 0.05 was considered statistically significant.

## 3 RESULTS

### 3.1 Pathological features of bAVM after partial embolization

**FIGURE 1:**
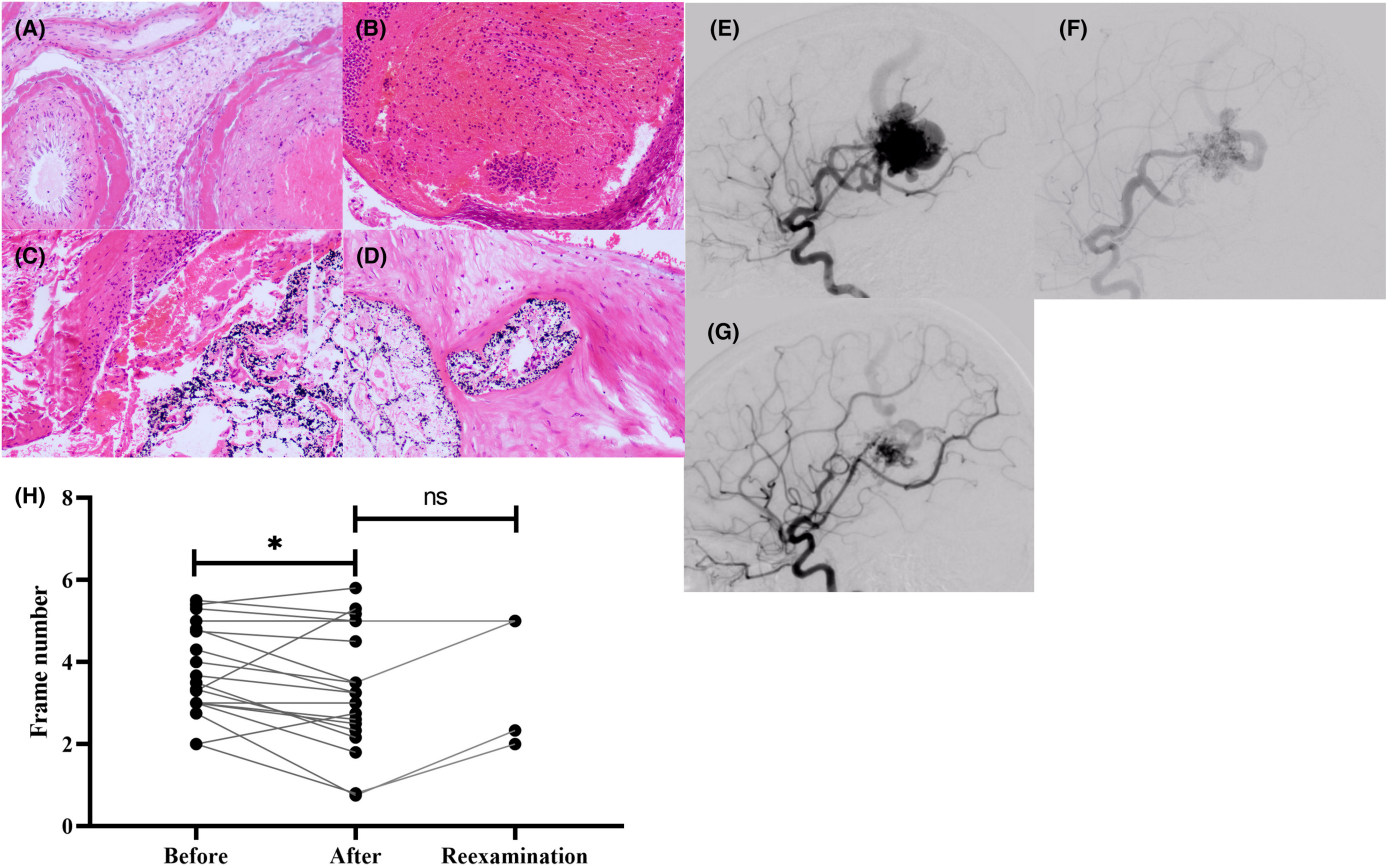
Results of H&E staining and bAVM nidal blood flow analysis. (A) Image of bAVM tissues, HE staining 100×. (B–D) Tissues image of bAVM after partial embolization at different times. (E–G) DSA image of patient no. 18. (E) When the draining vein is visualized, the peripheral vessels have entered the capillary phase. (F) The image just after partial embolization. When the draining vein is visualized, the peripheral vessels remain in the early arterial phase. (G) The image of 85 days after partial embolization. When the draining vein is visualized, the peripheral vessels have entered the late arterial phase. (H) The paired test results for hemodynamic change.

Malformed vessels, which have irregular vessel walls, were observed in all bAVM tissue sections, and some sections showed concomitant retention of embolic agents (Figure 1A,B,C,D). Specimens in the short‐term group showed numerous fresh thrombi in the embolized blood vessels, and inflammatory cells in the lumen (Figure 1B). The medium‐term group had an organized thrombus in the lumen and fewer infiltrating inflammatory cells, while significant fibrinous necrosis was observed in the layer of the vessel wall (Figure 1C). In the long‐term group, we detected obvious recanalization of thrombus and some fresh red blood cells in the neovascularized lumen (Figure 1D). The pathological features were consistent with the previous reports.^10^

### 3.2 Hemodynamic changes after partial embolization of bAVM

The majority of patients showed accelerated nidal blood flow after partial embolization (before partial embolization 3.76 ± 1.11 frames; after partial embolization 3.08 ± 1.64 frames; *p* = 0.0023) (Table 2). However, there was no statistically significant difference in the hemodynamic parameters before resection compared with data of patients after partial embolization (after partial embolization 3.08 ± 1.64 frames; reexamination before surgery 3.58 ± 1.64 frames; *p* = 0.18).Figure 1E,F,G presents a typical DSA image of a patient in the long‐term group.

**TABLE 2:** Hemodynamic changes after partial embolization.

Patients	Before partial embolization (frame number)	After partial embolization (frame number)	Reexamination before surgery (frame number)
Short‐term group			
1	3.50 ± 0.92	2.16 ± 0.75	/
2	3.00 ± 0.81	2.60 ± 0.54	/
3	5.50 ± 0.71	5.17 ± 0.75	/
4	3.00	2.50 ± 0.58	/
5	5.30 ± 0.58	5.00 ± 1.54	/
6	5.40 ± 0.55	5.80 ± 1.64	/
7	3.00 ± 0.81	1.80 ± 0.44	/
8	3.30 ± 0.58	5.30 ± 0.58	/
9	4.30 ± 0.82	3.25 ± 0.50	/
Medium‐term group			
10	3.67 ± 0.52	3.25 ± 0.96	/
11	4.00 ± 0.71	3.50 ± 1.0	/
12	5.00	5.00	5.00
13	4.75 ± 0.50	4.5.0 ± 1.23	/
14	3.00	3.00	/
15	2.00 ± 1.00	2.75 ± 0.50	/
16	3.33 ± 0.58	2.33 ± 0.58	/
17	/	/	/
Long‐term group			
18	2.75 ± 0.96	2.33 ± 1.03	2.83 ± 0.75
19	2.00	0.8 ± 0.84	2.00
20	4.80 ± 1.10	3.50 ± 0.58	5.00 ± 0.71
21	/	/	/
22	/	/	2.75 ± 0.96
23	/	/	3.50 ± 0.55
24	/	/	4.29 ± 0.49
Summary	3.76 ± 1.11	3.08 ± 1.64	3.58 ± 1.64
*p*‐Value		0.023*	0.180**

*Note*: / = Patient has missing DSA image. Values are expressed as the mean ± standard.

* Comparison between before partial embolization and after partial embolization.

** Comparison between before partial embolization and reexamination before surgery.

### 3.3 Inflammatory cell infiltration and endothelial cell‐to‐cell tight junctions in bAVM after partial embolization

**FIGURE 2:**
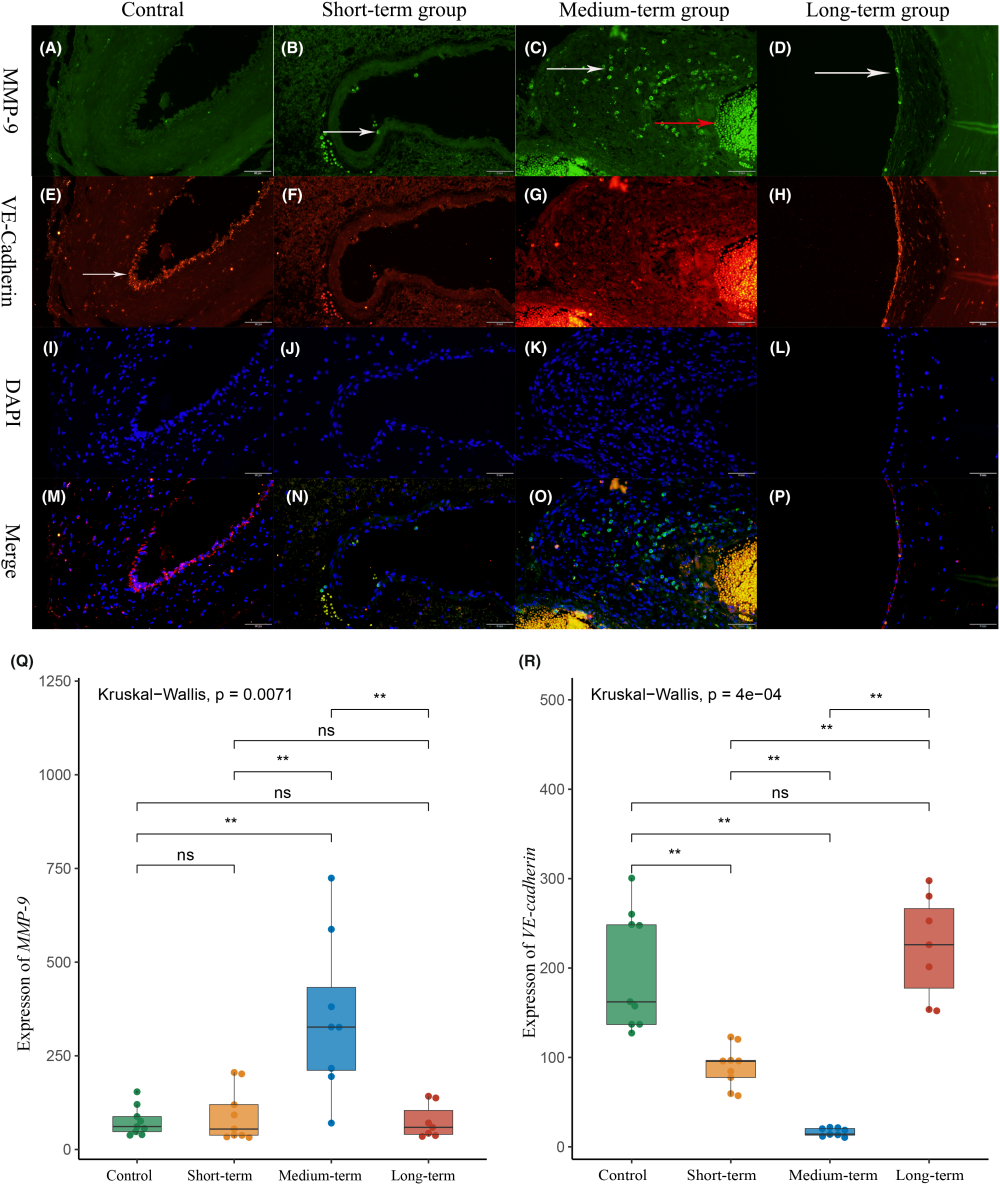
Inflammatory infiltration and cell junction integrity of bAVM malformation vessels after partial embolization, 200×. (A–D) MMP‐9 and biomarkers of inflammatory cells. (A) There was almost no inflammatory infiltration in the control group. (B–D) The vascular inflammatory infiltration in the short‐term and long‐term groups, with sporadic inflammatory cells attached to the vascular surface(white arrow). (C) A large number of inflammatory cells infiltrating the vessel wall in the metaphase group (white arrow) and the red arrow was non‐specific staining of red blood cells. (E–H) VE‐cadherin and biomarkers of cell junction. (E) The typical bAVM interendothelial cell junctions, with endothelial cell stacks and less continuous interendothelial cell junctions (white arrow). (F, G) Interendothelial cell junction almost disappeared in the short‐term and medium‐term groups, but it was relatively intact in the long‐term group (H). (I–L) DAPI and fluorescence of 4',6‐diamidino‐2‐phenylindole; (M–P) merge, merge of MMP‐P, VE‐cadherin, and DAPI. (Q, R) Quantitation of MMP‐9 and VE‐cadherin levels.*p<0.05; **p<0.01; ***p<0.005; ****p<0.001

Figure 2 shows the images of double‐immunostaining of MMP‐9 and VE‐cadherin. Quantitative results showed a significant infiltration of inflammatory cells in the medium‐term group compared with other groups (MMP‐9: medium‐term 353.50 ± 199.54 vs. short‐term 92.15 ± 69.90, *p* = 0.003; medium‐term vs. long‐term 59.04 (37.18, 137.57) *p* = 0.004; medium‐term vs. control 75.60 ± 39.30 *p* = 0.007; Figure 2Q,R) shows a semi‐quantitative analysis of VE‐cadherin. The integrity of endothelial cell‐to‐cell junctions in the short‐term and medium‐term groups were worse compared with the long‐term (VE‐cadherin: long‐term 223.42 ± 53.54 vs. short‐term 90.08 ± 21.87 *p* = 0.0025; long‐term vs. medium‐term 16.48 ± 4.25 *p* = 0.0078) and control groups (VE‐cadherin: control 198.62 ± 61.56 vs. short‐term *p* = 0.0025; control vs. medium‐term *p* = 0.0025). VE‐cadherin disappeared in the short‐term and medium‐term groups (Figure 2F,G). Interestingly, relatively intact interendothelial cell junctions were observed in the long‐term group (Figure 2H).

### 3.4 Proliferation in bAVM tissues after partial embolization

**FIGURE 3:**
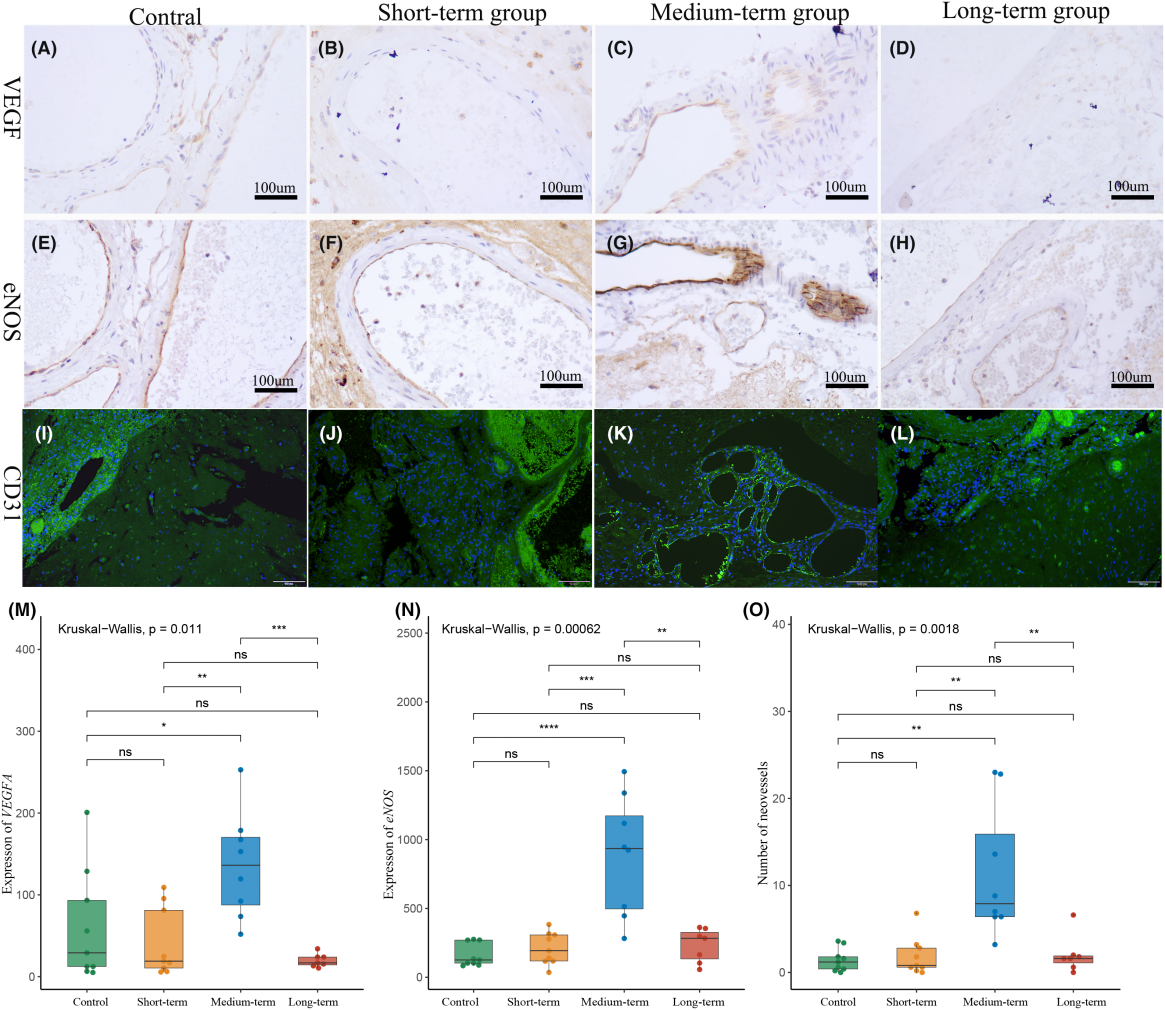
Increased expression of proliferation‐associated proteins in bAVMs after partial embolization (VEGFA, eNOS 200X; CD31, 100×). (A–D) VEGFA level of each group. (E–H) eNOS level of each group. (I–L) The neovessels increasing of bAVM after partial embolization. Green is the endothelial cell marker CD31 and DAPI is blue. (I, J) There were few neovessels in the control group and short‐term group. (K) Numerous neovessels are composed of a single layer of endothelial cells (yellow arrow). (L) The mature small vessel with an intact structure (red arrow) around the malformed vessel (white arrow). (M, N) The quantification of the mean IOD of VEGFA and eNOS. *p<0.05; **p<0.01; ***p<0.005; ****p<0.001. (O) The quantification of the mean neovessels number in each group. *p<0.05; **p<0.01; ***p<0.005; ****p<0.001

Figure 3 shows the result that the expression of angioproliferation‐associated proteins, VEGFA and eNOS, and the number of neovessels, in each bAVM sample. The analysis of IOD scores showed that the expression levels of VEGFA and eNOS in the medium‐term group were higher compared to those in the other groups (Figure 3M,N) (VEGFA: medium‐term 136.17 ± 61.16 vs. control 29.13 (9.435, 111.04) *p* = 0.0464; medium‐term vs. short‐term 18.95 (8.39, 88.30) *p* = 0.0055; medium‐term vs. long‐term 19.70 ± 7.51 *p* = 0.0003) (eNOS: medium‐term 882.72 ± 407.59 vs. Control 125.97 (95.63, 270.20) *p* < 0.0001; medium‐term vs. short‐term 209.69 ± 109.66, *p* = 0.0006; medium‐term vs. long‐term 231.63 ± 113.93 *p* = 0.0037). Counting the number of neovessels in each section based on CD31 staining. There were few malformed vessels in the control group or short‐term group (Figure 3I,J). However, in the medium‐term group (Figure 3K), the highest number of neovessels were detected (Figure 3O) (number of neovessels; medium‐term 7.90 (6.40, 18.20) vs. control 1.42 ± 1.25 *p* = 0.0013; medium‐term vs. short‐term 0.80 (0.40, 3.00), *p* = 0.002; medium‐term vs. long‐term 1.60 (0.60, 2.00) *p* = 0.0045), which concentrated around the malformed vessels. Figure 3K is a typical image. In the long‐term group, mature small vessels could be observed, with a complete vessel wall structure around the malformed vessels, while neovascularization were rarely encountered (Figure 3L).

### 3.5 Apoptosis in bAVM tissues after partial embolization

**FIGURE 4:**
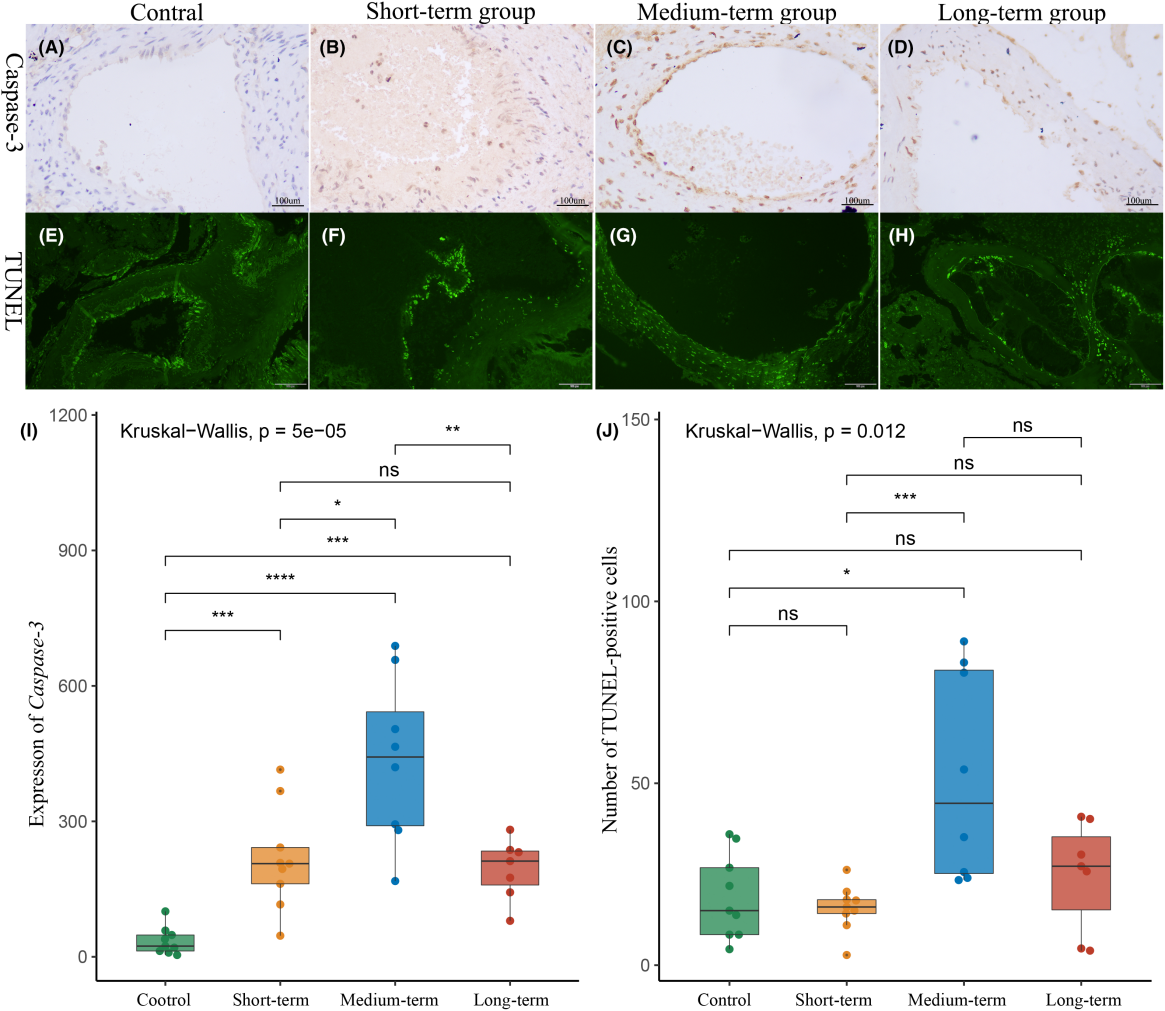
Apoptosis in bAVM tissues after partial embolization (Caspase‐3,200X; Tunel stain, 100×). (A–D) Caspase‐3 level of each group. (A, B) In the control group and short‐term group, caspase‐3 was found only in vascular endothelial cells. (C, D) After 24 h, caspase‐3 was highly expressed not only in vascular endothelial cells in bAVM tissue but also in vascular smooth muscle cells and fibroblasts. (E–H) Typical pictures of TUNEL staining of each group. (E, F) As same as caspase‐3, in the control and short‐term groups, TUNEL‐positive cells were mainly located in the endothelium. (G, H) TUNEL‐positive cells were expressed in both endothelium and vessel wall in the medium‐term and long‐term groups. (I) Quantification of the mean IOD of caspase‐3. (J) The numbers of the mean TUNEL‐positive cells in each group. *p<0.05; **p<0.01; ***p<0.005; ****p<0.001

Figure 4 shows the caspase‐3 IHC results and TUNEL staining results of bAVM tissue. Quantitative analysis showed that the expression of caspase‐3 in bAVM after embolization was significantly higher than that in the control group (Figure 4I) (caspase‐3; control 35.15 ± 25.85 vs. short‐term 217.47 ± 108.09 *p* = 0.0003; control vs. medium‐term 434.67 ± 171.41 *p* < 0.0001; control vs. long‐term 192.41 ± 62.40 *p* = 0.0003). Besides, caspase‐3 expression was the highest in the medium‐term group (Figure 4I) (caspase‐3; medium‐term vs. short‐term *p* = 0.0111; medium‐term vs. long‐term *p* = 0.0093). At the organizational level, TUNEL‐positive cells could be detected in all groups (Figure 4E,F,G,H ). Quantitative analysis indicated that the number of TUNEL‐positive cells was significantly elevated in the medium‐term group compared with the control and short‐term groups (Figure 4J) (TUNEL‐positive cells; medium‐term 51.83 ± 26.77 vs. control 18.82 ± 10.99 *p* = 0.0141; medium‐term vs. short‐term 15.69 ± 6.07 *p* = 0.0006). Although the number of TUNEL‐positive cells increased in the medium‐term group compared with that in the long‐term group, the difference was not statistically significant (TUNEL‐positive cells; medium‐term 51.83 ± 26.77 vs. long‐term 24.71 ± 14.00 *p* = 0.055).

## 4 DISCUSSION

Our study is the first to systematically describe the changes in vascular stability biomarkers of bAVM after partial embolization. We analyzed hemodynamics, cell junctions, inflammatory infiltration, proliferation, and apoptosis. These changes evolve dynamically over time. Compared with our study, previous work has only studied the changes in a single stability biomarker.^11^ We provide the first evidence that these vascular stability‐related indexes are dynamic, varying with the time between embolization and resection.

In our study, immediate alteration of the nidal blood flow after partial embolization was found. Consistent with our findings, previous studies have shown that residual nidal vessel blood flow velocity of bAVMs increased after partial embolization, and the blood redistribution to the non‐embolized AVM feeders could be observed.^12^ The immediate hemodynamic alteration may be related to the poor self‐regulation of malformed vessels.^13^ Hemodynamic changes may lead to vascular inflammation and this relationship has been well established in cerebral aneurysms.^14^ We also found a more severe inflammatory infiltration in the short‐term group than that in the control group. Hemodynamic changes and inflammation can cause both angiogenesis and vascular cell apoptosis. But, importantly, the angiogenesis in the short‐term group did not significantly differ from that in the control group, which is in contrast to earlier findings.^15^, ^16^ This discrepancy may be related to the fact that the preoperative interval time in the short‐term group was very short (3 h). Thus, the nidus could not fully respond to abruptly altered hemodynamic stress. An in vitro experiment revealed that VEGF‐related mRNA expression significantly increased in cerebral microvascular endothelial cells in response to the rapid increase of fluid shear stress.^17^ Recently, *Russo* et al. found that VEGFA expression was significantly up‐regulated in vascular endothelial cells after 24 h of exposure to pathological mechanical forces.^18^ These findings suggest that a certain period of time is necessary for angiogenesis in response to the altered hemodynamics. Apoptosis is also influenced by hemodynamics and inflammation. Caspase‐3 promotes the typical apoptosis features, including DNA fragmentation and cell death in several tissues.^19^ A previous study showed that vascular apoptosis in the endothelium is sensitive to disturbed blood flow, and disturbed blood flow could highly induce apoptotic endothelial micro‐particles after 30 min.^20^ Other studies have also confirmed that significant apoptosis, defined as intracellular DNA fragmentation, could only be observed in the presence of hemodynamic alterations for more than 24 h.^21^ It may explain the mismatch of caspase‐3 expression and the number of TUNEL‐positive cells in the short‐term group. Collectively, these results indicated that the vascular stability of bAVMs immediately changed after partial embolization, while this variation was not highly significant.

All indices of instability played significant roles in the medium‐term group. *Buell* et al. demonstrated that partial embolization could induce overexpression of hypoxia‐inducible factor 1‐alpha (HIF‐1α), VEGF, and MMP‐9 in bAVM tissues after 24 h.^15^ We also observed significant subendothelial inflammatory cell infiltration and disruption of tight junctions by double‐immunostaining of VE‐cadherin and MMP‐9. Consistent with previous studies, we found the up‐regulated expressions of VEGFA and eNOS, as well as the increased number of neovessels. The results indicated that vascular proliferation was promoted. This is not a good phenomenon for bAVM patients, because new capillaries lack a normal wall structure and are prone to hemorrhage.^22^ Few studies have concentrated on the apoptosis in bAVM after partial embolization, and we, in the present study, observed that caspase‐3 expression and the number of TUNEL‐positive cells were significantly elevated in the medium‐term group, which could be related to the altered hemodynamics and inflammatory infiltration.^23^ Importantly, a large number of TUNEL‐positive cells were distributed in both malformed vascular endothelium and smooth muscle, while it only was confined to endothelium in the short‐term group. The alteration of protein localization could be associated with the different levels of sensitivity of tissues to hemodynamic changes.^24^

A previous study reported the recanalization of vascular endothelial cells in bAVMs after embolization.^10^ In line with the literature, we, in the current study, found a significant recanalization in the long‐term group by H&E staining. And we also observed some intact small vessels, while few neovascular cells were identified by CD31 staining. Apparently, mature vessels are more stable than neovascularity without complete basal lamina.^6^ For the hemodynamic alterations in the long‐term group, although the sample size was small, we found a decline in residual nidal blood flow a few weeks after partial embolization. The aforementioned findings suggest that the neovascularity completed remodeling and the now mature blood vessels could reduce the nidal blood flow velocity by shunting blood, and the residual bAVM could reach a new equilibrium state. This hypothesis is similarly supported by the results of double‐immunostaining of VE‐cadherin and MMP‐9, and we only observed sporadic inflammatory cells attached to the vascular surface and more intact interendothelial cell junctions in the long‐term group.

## 5 LIMITATIONS

The present study has several limitations. First, as a retrospective study, we could not control the technical details of partial embolization, such as the degree of embolization and embolizing materials. However, previous research confirmed that the difference in the degree of embolization and embolic materials does not significantly impact the pathological of bAVMs.^7^ Second, the sample size was small. Given the rarity of bAVMs, increasing the sample size is difficult. In the future, multicenter joint studies may address this issue.

## 6 CONCLUSION

The biomarkers for bAVM vascular stability were most abnormal between 1 and 28 days after partial embolization, suggesting that bAVMs undergo the most nidal remodeling during this period. This may be a plausible explanation for the highest risk of rupture in the relatively early period after partial embolization in patients with bAVMs. Therefore, based on our results, we tend to support the viewpoint that resection surgery after partial embolization of bAVM patients should be performed early to avoid the high rupture risk period after partial embolization.

## FUNDING INFORMATION

This work was supported by the following grants: the Scientific and Technological Project of Henan Provincial Health Commission (No. SB201901068).

## CONFLICT OF INTEREST STATEMENT

None.

## Supporting information


Appendix S1.

## Data Availability

All data provided in this study are available from the corresponding author upon a reasonable requirement.
