# Supplementary material for: Vascular stability of brain arteriovenous malformations after partial embolization
Source: CNS Neurosci Ther. 2023 Feb 27;30(3):e14136. doi: 10.1111/cns.14136 (PMC10915995; doi:10.1111/cns.14136)
Supplement: Supplementary file 1 — Appendix S1. [file CNS-30-e14136-s001.docx]

**Method Details**

**Immunofluorescence and TUNEL staining**

Deparaffinization, rehydration, and antigen retrieval were conducted as same as immunohistochemistry. Tissue sections were blocked in QuickBlock™ blocking buffer (Beyotime) for 1 h at 37 ℃. Sections were then incubated with the following primary antibodies at 4 °C: anti-CD31 (1:100; ab28364; Abcam, Cambridge, UK), anti-MMP-9 (1:300; ab76003; Abcam), and anti-VE-cadherin (1:300; 66804-1-Ig; Proteintech, Rosemont, IL, USA). After rinsing with PBST, sections were incubated with secondary antibody (FITC-labeled goat anti-rabbit IgG (Beyotime) or Cy3-labeled goat anti-mouse IgG (Beyotime)) for 1 h at 37 ℃, followed by utilization of 4', 6-diamidino-2-phenylindole (DAPI) to label nuclear DNA of cells. Following completion of immunodetection, histological sections, which need to be examined for any length of time or to be stored, would be mounted under a cover-slip (Beyotime). The TUNEL assay was carried out using a TUNEL Apoptosis Detection kit (Abkine, Beijing, China). Images were captured using an Olympus BX43 Upright microscope. Representative images were analyzed with Image-Pro plus 6.0 software. The number of TUNEL-positive cells was counted by ImageJ software.

**Immunohistochemistry**

For IHC antigen retrieval, all sections were boiled in the Tris-EDTA buffer (1 mM, pH 9) for 20 min in a conventional microwave. Samples were allowed to cool to room temperature before incubation with 3% H_2_O_2_ for 15 min to neutralize endogenous peroxidase activity. After rinsing with PBST, tissue sections were blocked with 5% normal goat serum (ZSGB Biotechnology Co. , Ltd. , Shanghai, China) for 40 min at 37 ℃. Subsequently, sections were incubated overnight at 4 ℃ with primary antibodies, including anti-vascular endothelial growth factor A (VEGFA) (1:150; ab52917; Abcam), anti-endothelial nitric oxide synthase (eNOS (1:200; ab252439; Abcam), and anti-caspase-3 (1:150; 66470-2-Ig; Proteintech). Sections were then washed with phosphate-buffered saline (PBS) and incubated with horseradish peroxidase-labeled goat anti-rabbit or anti-mouse antibody (ZSGB Biotechnology Co, Ltd. ) for 40 min at room temperature. Staining was completed by a 3 min incubation with DAB substrate-chromogen buffer before counterstaining with hematoxylin. An Olympus microscope and a digital color camera (E3ISPM2000; ToupTek, Beijing, China) were used to visualize and to photograph the slides, respectively. Five images were randomly taken for analysis of each section of each patient. For each image, 3 random vascular areas without embolic agents were picked to calculate the IOD. The expressions of all target proteins were assessed as IOD scores. The IOD scores of all target proteins were calculated using Image-Pro plus 6.0 software.
